# *In vitro* and *In vivo* Assessment of Suitable Reference Region and Kinetic Modelling for the mGluR1 Radioligand [^11^C]ITDM in Mice

**DOI:** 10.1007/s11307-019-01435-1

**Published:** 2019-12-02

**Authors:** Daniele Bertoglio, Jeroen Verhaeghe, Špela Korat, Alan Miranda, Leonie wyffels, Sigrid Stroobants, Ladislav Mrzljak, Celia Dominguez, Longbin Liu, Mette Skinbjerg, Ignacio Munoz-Sanjuan, Steven Staelens

**Affiliations:** 1 Molecular Imaging Center Antwerp (MICA), Faculty of Medicine and Health Sciences, University of Antwerp, Universiteitsplein 1, Wilrijk, Belgium; 2 Department of Nuclear Medicine, Antwerp University Hospital, Edegem, Belgium; 3 CHDI Management/CHDI Foundation, Los Angeles, CA USA

**Keywords:** Kinetic modelling, mGluR1, [^11^C]ITDM, PET, Reference region, Mouse, Arteriovenous shunt, Preclinical imaging

## Abstract

**Purpose:**

This study aimed at investigating binding specificity, suitability of reference region-based kinetic modelling, and pharmacokinetics of the metabotropic glutamate receptor 1 (mGluR1) radioligand [^11^C]ITDM in mice.

**Procedures:**

We performed *in vivo* blocking as well as displacement of [^11^C]ITDM during positron emission tomography (PET) imaging using the specific mGluR1 antagonist YM-202074. Additionally, we assessed *in vitro* blocking of [^3^H]ITDM at two different doses of YM-202074. As an alternative to reference region models, we validated the use of a noninvasive image-derived input function (IDIF) compared to an arterial input function measured with an invasive arteriovenous (AV) shunt using a population-based curve for radiometabolite correction and characterized the pharmacokinetic modelling of [^11^C]ITDM in the mouse brain. Finally, we also assessed semi-quantitative approaches.

**Results:**

*In vivo* blocking with YM-202074 resulted in a decreased [^11^C]ITDM binding, ranging from − 35.8 ± 8.0 % in pons to − 65.8 ± 3.0 % in thalamus. Displacement was also markedly observed in all tested regions. In addition, *in vitro* [^3^H]ITDM binding could be blocked in a dose-dependent manner. The volume of distribution (*V*_T_) based on the noninvasive IDIF (*V*_T (IDIF)_) showed excellent agreement with the *V*_T_ values based on the metabolite-corrected plasma input function regardless of the metabolite correction (*r*^2^ > 0.943, *p* < 0.0001). Two-tissue compartmental model (2TCM) was found to be the preferred model and showed optimal agreement with Logan plot (*r*^2^ > 0.960, *p* < 0.0001). A minimum scan duration of 80 min was required for proper parameter estimation. SUV was not reliable (*r*^2^ = 0.379, *p* = 0.0011), unlike the SUV ratio to the SUV of the input function, which showed to be a valid approach.

**Conclusions:**

No suitable reference region could be identified for [^11^C]ITDM as strongly supported by *in vivo* and *in vitro* evidence of specific binding in all brain regions. However, by applying appropriate kinetic models, [^11^C]ITDM PET imaging represents a promising tool to visualize mGluR1 in the mouse brain.

**Electronic supplementary material:**

The online version of this article (10.1007/s11307-019-01435-1) contains supplementary material, which is available to authorized users.

## Introduction

Glutamate, the major excitatory neurotransmitter in the brain, plays an essential role in a variety of physiological processes. Among several ionotropic and metabotropic receptors, the metabotropic glutamate receptors (mGluRs) are G protein-coupled receptors involved in the modulation of synaptic transmission and neuronal excitability [1]. The mGluRs of the group I are located post-synaptically, and they include the mGluR type 1 (mGluR1) and type 5 (mGluR5).

Both mGluR1 and mGluR5 have been linked to a number of neurological disorders, including epilepsy, stroke, fragile X syndrome, Huntington’s disease, obsessive-compulsive disorder, Alzheimer’s disease, Parkinson’s disease, and drug addiction [2]. Thus, given the relevance of group I mGluRs for the evaluation of potential therapeutic interventions, there is a growing interest for *in vivo* monitoring of group I mGluRs in the living brain which can be achieved by means of positron emission tomography (PET) imaging. Although mGluR1 and mGluR5 share a high degree of homology, they are characterized by a distinct cerebral expression pattern, with mGluR5 mainly distributed in the striatum, hippocampus, and cortex, whereas mGluR1 primarily located in the thalamus and in the cerebellum [3, 4].

While a large body of literature on preclinical and clinical mGluR5 PET imaging is available [5], only a limited number of studies in disease models have been reported for mGluR1 PET imaging [6]. In particular, the application of mGluR1 PET imaging in the mouse brain has been extremely limited to date, an important shortcoming given the relevance of mouse models in understanding the pathophysiology of neurological disorders.

Among the radiotracers for mGluR1 described in the literature, N-[4-[6-(isopropylamino)-pyrimidin-4-yl]-1,3-thiazol-2-yl]-N-methyl-4-[^11^C]methylbenzamide ([^11^C]ITDM) [7] is one of the most promising and better characterized radiotracers in the preclinical (rats and rhesus monkeys) settings [7–9]. Notably, Yamasaki and colleagues [8] claimed that the pons is a suitable reference region for noninvasive kinetic modelling of [^11^C]ITDM in rats. [^11^C]ITDM has also been employed to investigate changes in mGluR1 levels in a mouse model of Huntington’s disease using the pons as reference region [8]. However, as *in vivo* validation has not been performed in mice, it is not clear yet whether the pons is a receptor-free region suitable as reference region. A proper validated reference region is of the utmost importance since erroneous selection of reference region for quantifying PET signal may lead to significant misinterpretation of PET data.

For these reasons, the first aim of the present study was to validate the specific binding of [^11^C]ITDM and to investigate whether a suitable a reference region exists in the mouse brain. To this end, we performed *in vitro* blocking of [^3^H]ITDM as well as *in vivo* blocking and displacement of [^11^C]ITDM with the specific mGluR1 antagonist YM-202074 [10]. Since invasive arterial blood sampling in mice presents several challenges and limitations in the perspective of longitudinal studies, the second aim of the study was to investigate whether an image-derived input function (IDIF) could be used as a valid alternative approach for noninvasive quantification of [^11^C]ITDM PET imaging in the eventual absence of a reference region. The third aim was to characterize suitable kinetic models for [^11^C]ITDM quantification and to assess possible semi-quantitative approaches.

## Materials and Methods

### Animals

Adult male C57BL/6J mice from Jackson Laboratories (Bar Harbour, ME, USA) were used in the study. Details of the animals are provided in the Electronic Supplementary Material (ESM).

### Radiotracer Synthesis

[^11^C]ITDM synthesis was performed on an automated synthesis module (Carbosynthon I, Comecer, The Netherlands) based on [7]. Details of the radiosynthesis are provided in the ESM.

### PET Acquisition

Two Siemens Inveon PET/CT scanners (Siemens Preclinical Solution, Knoxville, USA) were used to acquire the dynamic microPET/computed tomography (CT) images. Animal preparation was performed as previously described [11, 12]. Details of the PET acquisition are provided in the ESM.

### Image Reconstruction and Processing

Details of the image reconstruction and processing are provided in the ESM.

### Metabolite Correction

In order to generate a population-based metabolite correction to account for peripheral radiometabolism, we measured parent fractions in a cohort of WT mice (*n* = 3 per time point) at 5, 15, and 30 min p.i. The procedure was done adapting the previously described methodology [13] to [^11^C]ITDM. Details of the procedure are provided in the ESM.

### Kinetic Modelling

During the baseline and blocking study, regional TACs were fitted by the Logan plot method [14] using the noninvasive IDIF as previously validated [11]. This approach allows comparative studies  to be performed in mice, avoiding blood sampling, which is invasive and not feasible for longitudinal studies. To assess the extent of target occupancy reached during the blocking study with YM-202074, we applied the Lassen plot [15] based on the regional changes in *V*_T (IDIF, Uncorr)_ following drug pretreatment. Simultaneously, we evaluated whether future kinetic modelling could rely on a reference model. Parametric maps were generated through voxel-based graphical analysis (Logan plot) with the IDIF (Uncorr) as input function.

In absence of a suitable reference region, full kinetic modelling was now performed by fitting the regional TACs by one-tissue compartmental model (1TCM), two-tissue compartmental model (2TCM), and Logan plot in order to estimate the total volume of distribution *V*_T (AV shunt, Corr)_ as well as its noninvasive surrogate *V*_T (IDIF, Uncorr)_ using the noninvasive IDIF. The blood volume fraction (*V*_B_) was fixed at 3.6 % [16] for the compartmental models, while, for the Logan plot, the linear phase (t*) was calculated from the curve fitting with t* ranging 12.5–20 min depending on the brain region.

Details of the model selection, stability of outcome parameters, and assessment of simplified approaches are provided in the ESM.

### Brain Tissue Collection

Animals were euthanized by decapitation while under anaesthesia and brains were snap-frozen in 2-metylbuthane at − 35 °C for 2 min and further preserved at −80 °C until use. Sagittal sections (20 μm of thickness) were collected starting at 0.96 mm lateral according to Paxinos and Franklin [17] in triplicate on Superfrost Plus slides (Thermo Fischer Scientific, USA), using a cryostat (Leica, Germany).

### [^3^H]ITDM Autoradiography

To further evaluate the suitability of a reference region, we investigated the effect of mGluR1 blockade on *in vitro* [^3^H]ITDM autoradiography. Details of the procedure are provided in the ESM.

### Statistical Analysis

Repeated measurements two-way ANOVA was applied to compare *in vivo* PET and *in vitro* autoradiography values between baseline and blockade in different regions as well as to evaluate differences between *V*_T_ values based on AV shunt and IDIF as input functions. Pearson’s correlation tests were used when assessing relationship between two variables. Pearson’s correlation tests as well as Bland-Altman plots were applied to compare *V*_T (AV shunt, Corr)_ and *V*_T (IDIF, Uncorr)_ values determined with 2TCM and Logan plot. Analyses were performed with GraphPad Prism (v 6.0) statistical software. The data are represented as mean ± standard deviation (SD), unless specified otherwise. All tests were two-tailed and significance was set at *p* < 0.05.

## Results

### Assessment of [^11^C]ITDM Specific Binding Demonstrates Lack of Suitable Reference Region

**Fig. 1.**
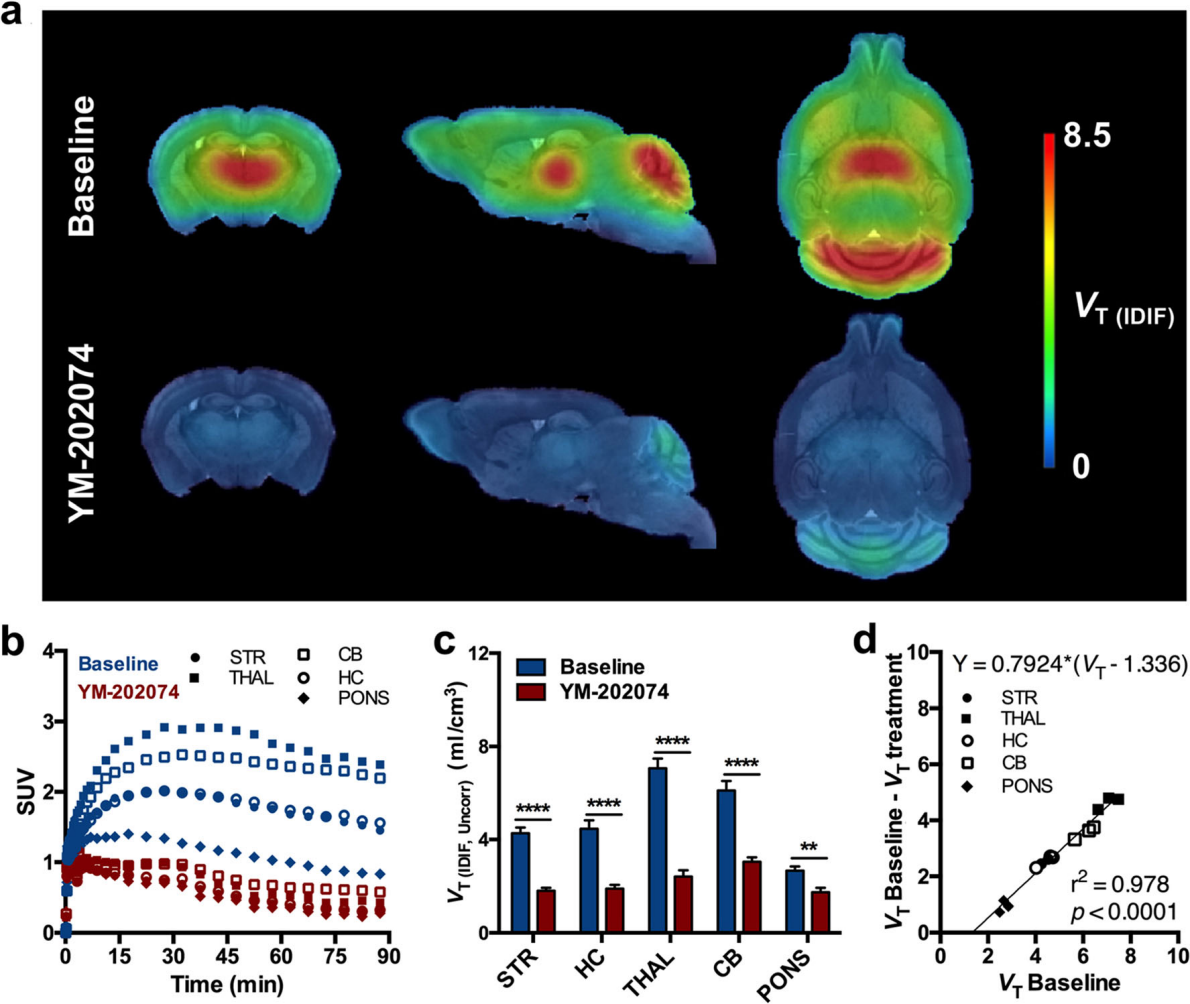
Effect of *in vivo* blockade on [^11^C]ITDM binding. **a** Average parametric maps during baseline and pretreatment with YM-202074 (20 mg/kg, i.v.) overlaid onto a MRI mouse brain template for anatomical localization. **b** SUV time-activity curves and **c ***V*_T (IDIF, Uncorr)_ quantification during baseline and pretreatment with YM-202074. **d** Lassen graphical analysis estimated a mGluR1 fitted occupancy of 79 %. **p < 0.01, ****p < 0.0001. *n* = 3/group. STR, striatum; THAL, thalamus; HC, hippocampus; CB, cerebellum.

To confirm the specificity of [^11^C]ITDM for mGluR1 and determine whether reference region-based kinetic models could be applied in the mouse brain, first we performed *in vivo* blocking of [^11^C]ITDM with the mGluR1 antagonist YM-202074. [^11^C]ITDM parametric *V*_T (IDIF,Uncorr)_ maps of baseline and blockade scans are shown in Fig. 1a. SUV TACs during baseline scan displayed the peak of radioactivity around 30 min post-injection, with the highest uptake in cerebellum and thalamus, followed by a slow wash-out (Fig. 1b). Pretretament with YM-202074 (20 mg/kg, i.v. 2 min prior to [^11^C]ITDM injection) resulted in an evident decline in all the investigated regions demonstrating a marked blockade of mGluR1. Hence, *V*_T (IDIF,Uncorr)_ values during pretreatment with YM-202074 were significantly reduced (thalamus, cerebellum, striatum, and hippocampus: *p* < 0.0001; pons: *p* < 0.01) compared to baseline as shown in Fig. 1c. *V*_T (IDIF,Uncorr)_ values during baseline and pretreatment with YM-202074 for each region are reported in Table 1. The largest difference in *V*_T (IDIF, Uncorr)_ values was found in thalamus (baseline, 7.06 ± 0.42 ml/cm^3^; YM-202074, 2.41 ± 0.27 ml/cm^3^; − 65.8 ± 3.0 %, *p* < 0.0001), while the smallest in pons (baseline, 2.66 ± 0.18 ml/cm^3^; YM-202074, 1.73 ± 0.19 ml/cm^3^; − 35.8 ± 8.0 %, *p* < 0.01). Additionally, by applying the Lassen graphical analysis, we could estimate that pretreatment resulted in a mGluR1 occupancy by YM-202074 of 79.2 % (Fig. 1d).

**Table 1:** Effect of mGluR1 blockade on [^11^C]ITDM binding. *V*_T (IDIF, Uncorr)_ quantification during baseline and pretreatment with YM-202074 determined by Logan plot based on 90 min acquisition

Region	Baseline	Pretreatment with YM-202074	
	*V*_T_ (ml/cm^3^)		
	Mean (SD)	Mean (SD)	Decrease (%)
Striatum	4.27 (0.25)	1.80 (0.12)	− 57.8****
Thalamus	7.06 (0.42)	2.41 (0.27)	− 65.8****
Hippocampus	4.46 (0.37)	1.89 (0.16)	− 57.6****
Cerebellum	6.11 (0.41)	3.04 (0.19)	− 50.2****
Pons	2.66 (0.18)	1.73 (0.19)	− 34.9**

*n* = 3

**p < 0.01, ****p < 0.0001

**Fig. 2.**
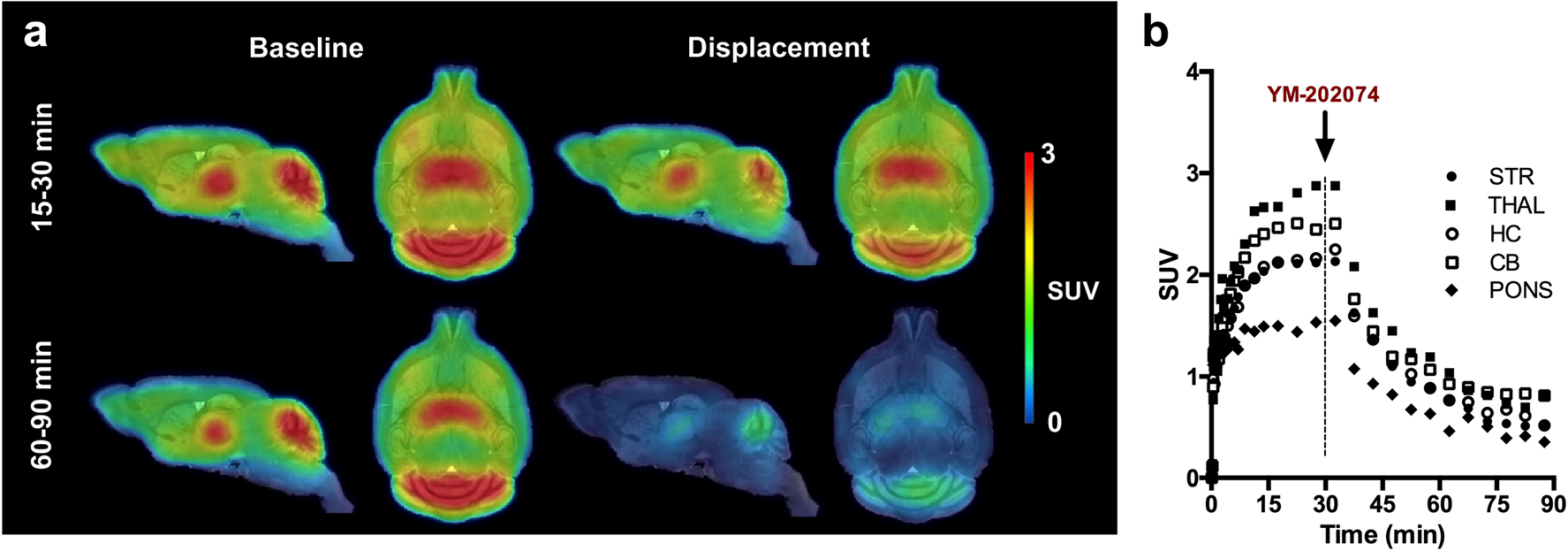
Displacement of [^11^C]ITDM. **a** SUV images based on 15–30 min and 60–90 min during baseline and displacement with YM-202074 (20 mg/kg, i.v.) injected 30 min after radiotracer. Images are overlaid onto a MRI mouse brain template for anatomical localization. **b** SUV time-activity curves indicated [^11^C]ITDM was displaced in all regions. Arrow indicates the injection of YM-202074. *n* = 3/group. STR, striatum; THAL, thalamus; HC, hippocampus; CB, cerebellum.

To further support these findings, an *in vivo* displacement study was performed by injecting YM-202074 (20 mg/kg, i.v.) 30 min following start of the scan. SUV summed PET images covering 15–30 min and 60–90 min post-radiotracer injection were generated for visual comparison (Fig. 2a). As shown in the SUV TACs (Fig. 2b), [^11^C]ITDM displacement after injection of YM-202074 resulted in a robust and manifest effect in the whole brain.

**Fig. 3.**
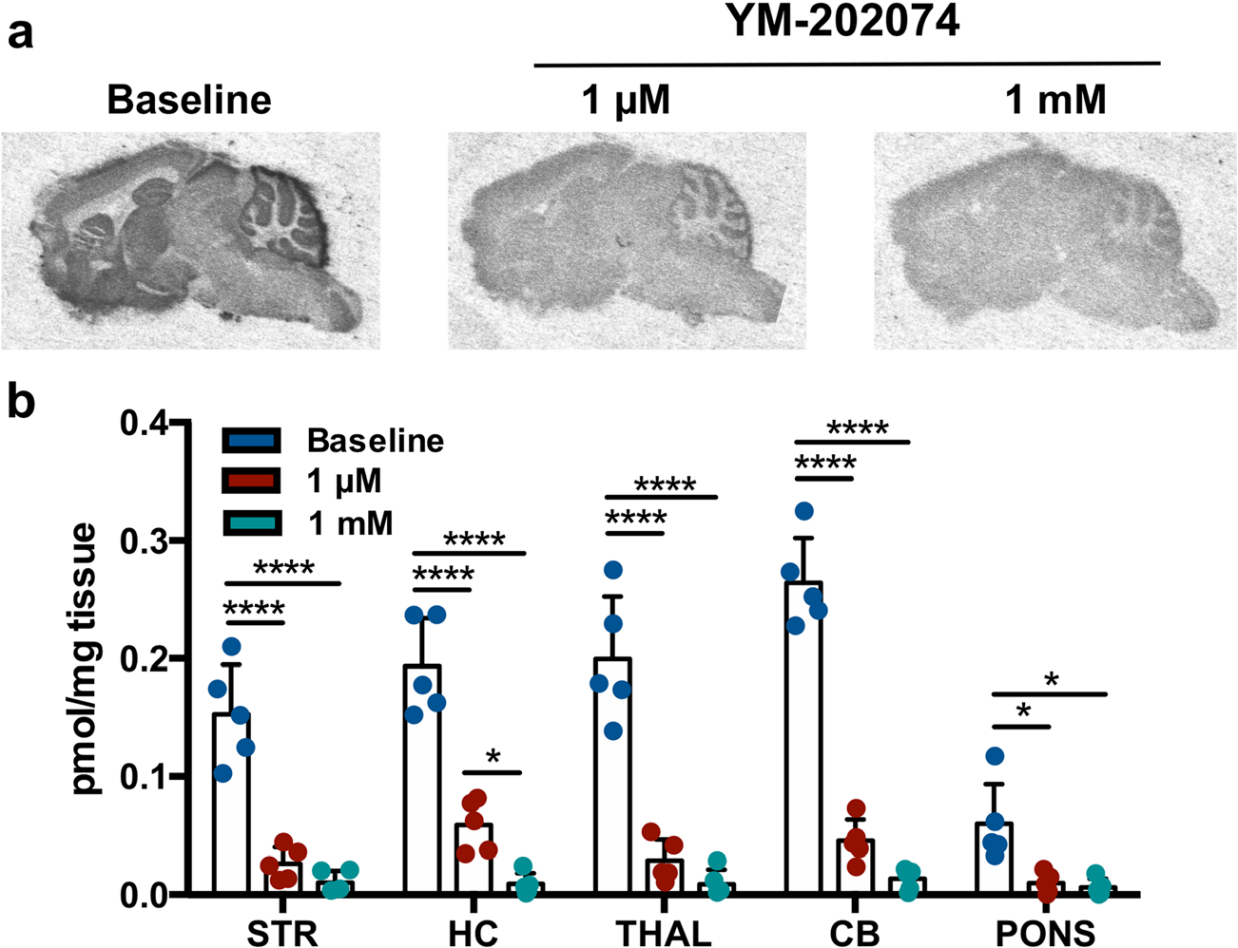
Effect of *in vitro* blockade on [^3^H]ITDM binding. **a** Representative autoradiograms of [^3^H]ITDM during baseline and blocking with YM-202074 in the same brain. **b** Quantification of [^3^H]ITDM for baseline and coincubation with YM-202074 (1 μM and 1 mM). *n* = 5/group. *p < 0.05, ****p < 0.0001. STR, striatum; THAL, thalamus; HC, hippocampus; CB, cerebellum.

Finally, we performed an *in vitro* blocking study with [^3^H]ITDM autoradiography. As shown in Fig. 3, coincubation of [^3^H]ITDM with YM-202074 resulted in a statistically significant decline in a dose-dependent manner in striatum, hippocampus, thalamus, cerebellum (*p* < 0.0001), and pons (*p* < 0.05).

### Image-Derived Input Function Allows Noninvasive *V*_T_ Quantification

Since no suitable reference region was present in the mouse brain, we investigated whether an IDIF is accurate to circumvent the need for an invasive input function which disables longitudinal studies.

**Fig. 4.**
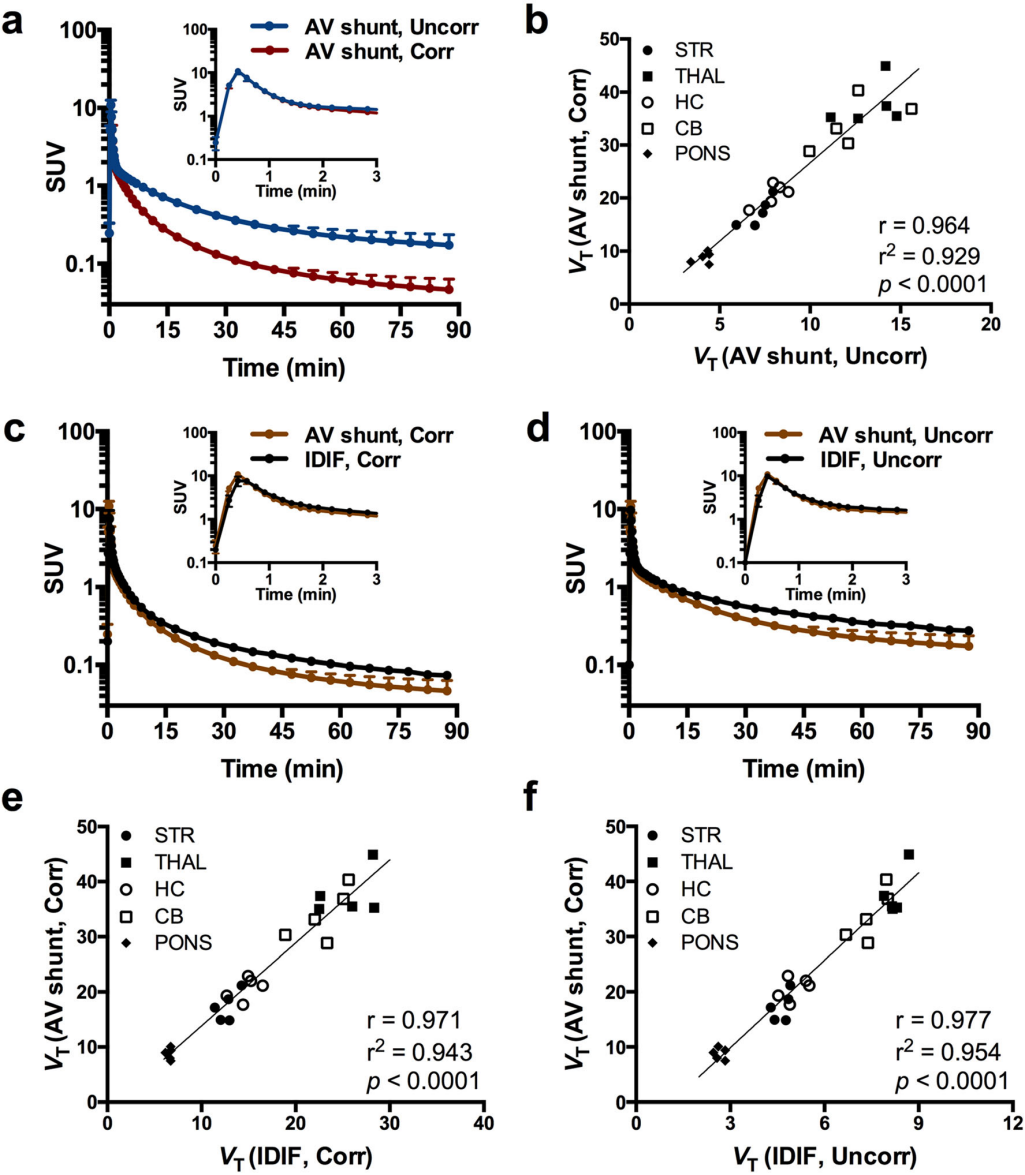
Comparison of invasive and noninvasive input functions for [^11^C]ITDM quantification. **a** Average plasma SUV time-activity curves (TACs) corrected and uncorrected for radiometabolism and **b** correlation between *V*_T (AV shunt)_ based on 2TCM using plasma input function corrected (AV shunt, Corr) and uncorrected for radiometabolism (AV shunt, Uncorr). **c** Comparison between average SUV TACs for invasive (AV shunt) and noninvasive (IDIF) input functions corrected and **d** uncorrected for plasma radiometabolism. Both *V*_T (IDIF)_ values based on **e** 2TCM corrected (IDIF, Corr) and **f** uncorrected (IDIF, Uncorr) for plasma radiometabolites showed excellent correlation with *V*_T (AV shunt, Corr)_ based on 2TCM using the metabolite-corrected plasma input function. Solid line represents the linear regression. Data are presented as mean ± SEM. *n* = 5. STR, striatum; THAL, thalamus; HC, hippocampus; CB, cerebellum.

In order to validate the noninvasive IDIF, *V*_T (IDIF, Corr)_ and *V*_T (IDIF, Uncorr)_ were estimated and compared to the *V*_T (2TCM)_ determined using the radiometabolite-corrected plasma activity input function based on the AV shunt measurement *V*_T (AV shunt, Corr)_. Average plasma SUV TACs corrected and uncorrected for radiometabolism are shown in Fig. 4a, with the derived *V*_T (AV shunt, Corr)_ and *V*_T (AV shunt, Uncorr)_ values displaying high agreement (*r* = 0.964, *r*^2^ = 0.929, *p* < 0.0001) (Fig. 4b). Comparison of the invasive (AV shunt) and noninvasive (IDIF) input functions revealed the same peak values; however, the IDIF tail values were higher compared to the equivalent invasive values either correcting (Fig. 4c) or not (Fig. 4d) for radiometabolites. Accordingly, noninvasive *V*_T (IDIF)_ values corrected (*V*_T (IDIF, Corr)_) and uncorrected (*V*_T (IDIF, Uncorr)_) for plasma metabolism highly correlated with the *V*_T (AV shunt, Corr)_ values (*r*^2^ = 0.971, *p* < 0.0001; and *r*^2^ = 0.977, *p* < 0.0001, respectively) with a linear relationship (Fig. 4e, f) indicating that the noninvasive *V*_T (IDIF)_ values were proportional to the invasive ones. Noteworthy, the use of the radiometabolite-corrected plasma IDIF did not improve the relation of *V*_T (IDIF,corr)_ to *V*_T (AV shunt, Corr)_; thus, the uncorrected IDIF could be used as noninvasive input function to determine *V*_T (IDIF, Uncorr)_ in future longitudinal studies.

### Pharmacokinetic Analysis of [^11^C]ITDM

Following evidence of the lack of a suitable reference region for [^11^C]ITDM and the validation of the noninvasive IDIF for *V*_T (IDIF, Uncorr)_ quantification, we evaluated several models (Logan plot, 1TCM, and 2TCM) for estimation of the *V*_T_ based on invasive radiometabolite-corrected plasma activity AV shunt (*V*_T (AV shunt, Corr)_) as well as the noninvasive *V*_T (IDIF, Uncorr)_. According to the AIC (model with the lowest value is the preferred), 2TCM was the desired model (Suppl. Table 1, see ESM), in agreement with the MSC (preferred model has the highest value) (Suppl. Table 2, see ESM). Thus, 1TCM was excluded as it did not fit the data as displayed in the representative fitting in Suppl. Fig. 3 (see ESM).

Both *V*_T (AV shunt, Corr)_ and *V*_T (IDIF, Uncorr)_ values determined using 2TCM and Logan plot based on 90 min acquisition resulted in high agreement (*V*_T (AV shunt, Corr)_: *r*^2^ = 0.960, *p* < 0.0001; *V*_T (IDIF, Uncorr)_: *r*^2^ = 0.986, *p* < 0.0001) without deviating from the identity line as shown in Suppl. Fig. 4 (see ESM). Accordingly, the Bland-Altman plots revealed only a negligible bias for both *V*_T (AV shunt, Corr)_ (− 4.97 %) and *V*_T (IDIF, Uncorr)_ (+ 0.15 %) (Suppl. Fig. 4, see ESM). The *in vivo* kinetic parameters of [^11^C]ITDM based on 2TCM and Logan plot using the metabolite-corrected plasma input function during 90 min acquisition are described in Table 2.

**Table 2:** [^11^C]ITDM pharmacokinetics. *In vivo* kinetic parameters of [^11^C]ITDM determined using 2TCM and Logan plot based on 90 min acquisition with metabolite-corrected plasma AV shunt as input function (*V*_T (AV shunt, Corr)_)

Region	2TCM						Logan	
	*K*_1_ (ml/cm^3^/min)	*k*_2_ (per min)	*k*_3_ (per min)	*k*_4_ (per min)	*V*_T_ (ml/cm^3^)		*V*_T_ (ml/cm^3^)	
	Mean (SD)	Mean (SD)	Mean (SD)	Mean (SD)	Mean (SD)	COV (%)	Mean (SD)	COV (%)
Striatum	0.53 (0.14)	1.24 (0.50)	0.84 (0.17)	0.020 (0.005)	17.3 (2.6)	15.4	15.9 (2.6)	16.5
Thalamus	0.73 (0.44)	1.02 (0.73)	0.91 (0.47)	0.016 (0.008)	37.6 (4.2)	11.1	30.0 (4.6)	15.4
Hippocampus	0.49 (0.13)	1.26 (0.51)	0.81 (0.15)	0.017 (0.005)	20.6 (2.1)	10.1	18.9 (2.1)	11.1
Cerebellum	0.67 (0.26)	1.33 (0.65)	0.95 (0.22)	0.012 (0.003)	33.9 (4.7)	13.9	31.5 (3.9)	12.4
Pons	0.63 (0.07)	1.10 (0.27)	0.49 (0.11)	0.025 (0.003)	8.8 (1.0)	11.8	8.5 (0.9)	10.5

*n* = 5

**Fig. 5.**
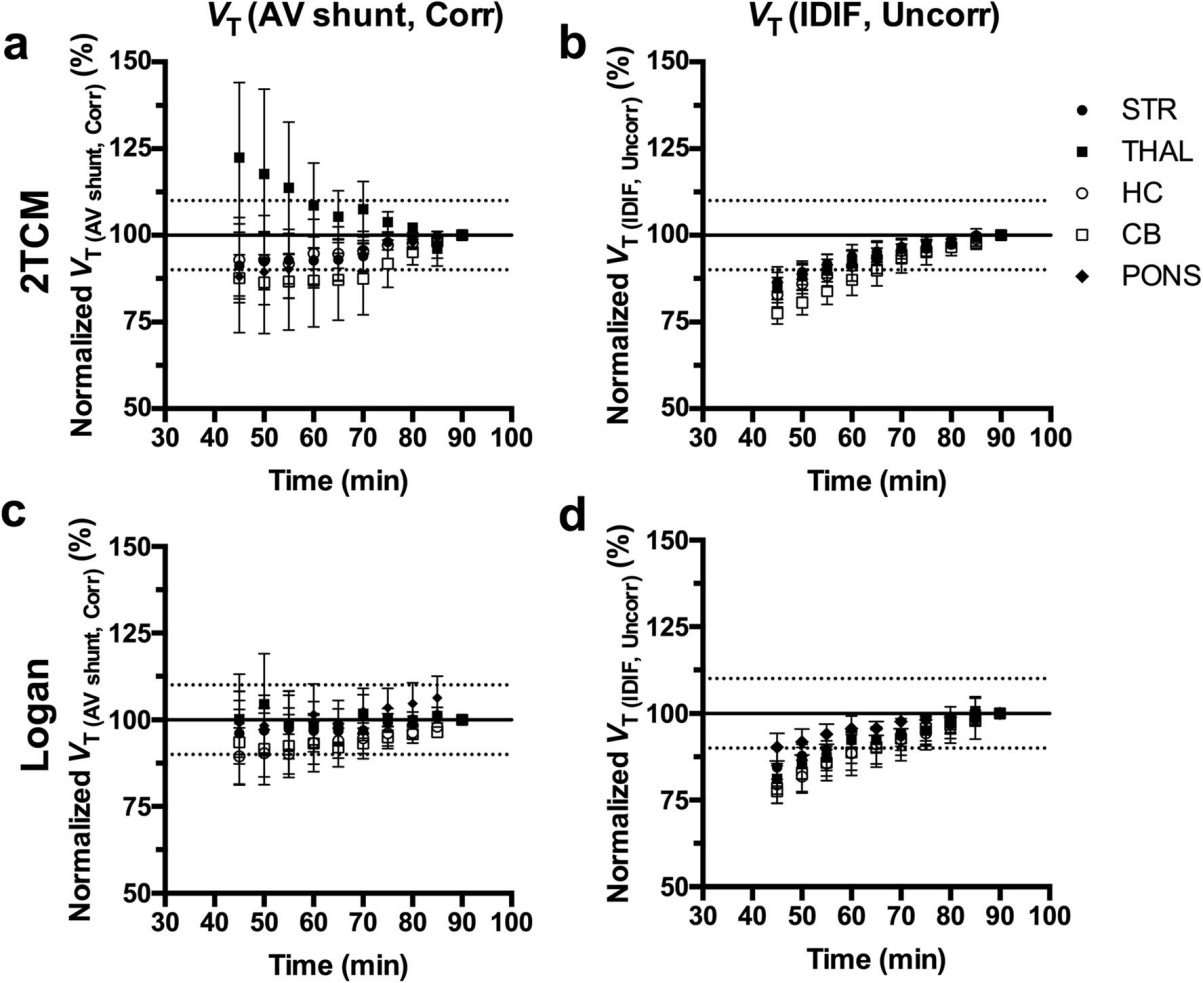
Time stability of parameter estimation in different brain regions. **a ***V*_T (AV shunt, Corr)_ and **b ***V*_T (IDIF, Uncorr)_ calculated using 2TCM and **c**, **d** Logan plot normalized to the values obtained during 90 min acquisition. *n* = 5. STR, striatum; THAL, thalamus; HC, hippocampus; CB, cerebellum.

Finally, we investigated the time stability of parameter estimation for both *V*_T (AV shunt, Corr)_ and *V*_T (IDIF, Uncorr)_. As depicted in Fig. 5, shortening the scan duration resulted in a large inter-individual variability of *V*_T (AV shunt, Corr)_ values based on both 2TCM and Logan plot, and an underestimation for *V*_T (IDIF, Uncorr)_ values. For instance, reducing the acquisition time to 60 min showed an average bias for cerebellum based on 2TCM of − 13.1 ± 13.2 % (Fig. 5a) and − 13.7 ± 4.8 % (Fig. 5b) for *V*_T (AV shunt, Corr)_ and *V*_T (IDIF, Uncorr)_, respectively. Similarly, Logan plot resulted in an average bias of − 6.8 ± 8.1 % (Fig. 5c) and − 11.2 ± 5.1 % (Fig. 5d) for *V*_T (AV shunt, Corr)_ and *V*_T (IDIF, Uncorr)_, respectively. Thus, an acquisition of 80 min or longer is required in order to obtain accurate parameter estimates.

### Assessment of Simplified Approaches for [^11^C]ITDM Measurement

**Fig. 6:**
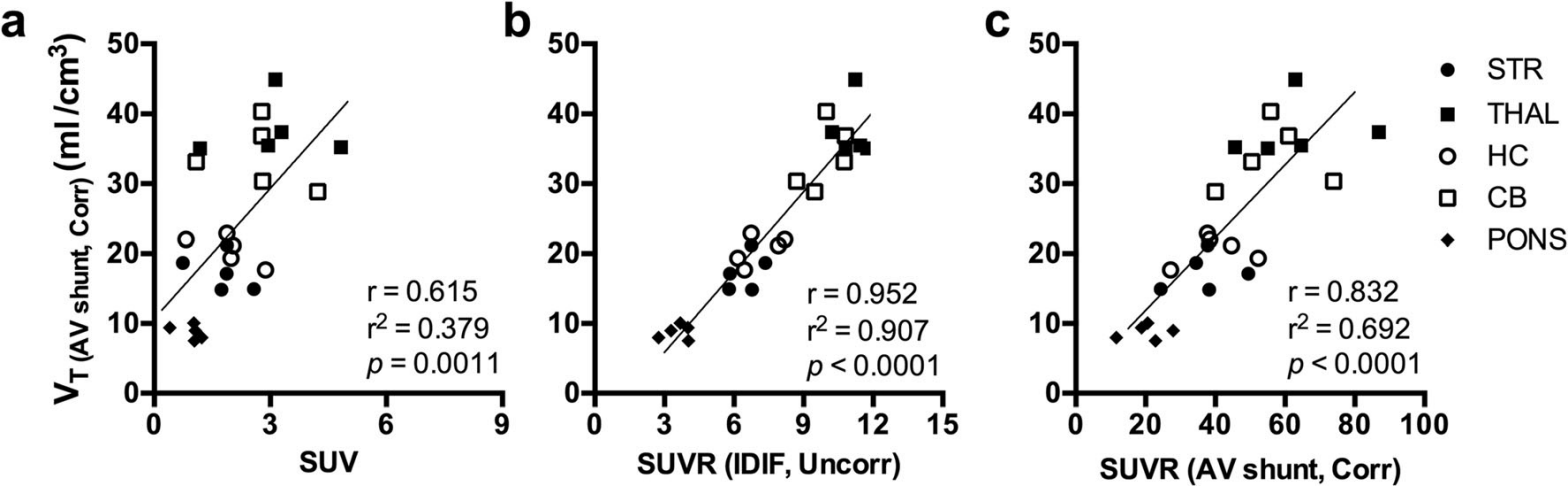
Assessment of simplified approaches for [^11^C]ITDM measurement. **a ***V*_T (AV shunt, Corr)_ values are plotted in relation to SUV (60–90 min), **b** SUVR (60–90 min) using as reference the SUV of the input function based on IDIF _(IDIF, Uncorr)_ or **c** AV shunt _(AV shunt, Corr)_. Dashed line depicts the identity line. *n* = 5. STR, striatum; THAL, thalamus; HC, hippocampus; CB, cerebellum; SUVR, SUV of tissue to reference input function.

Given the long PET acquisition required for parameter estimation, we explored whether a simplified approach for measurement of [^11^C]ITDM could be applied. Specifically, we compared the calculated *V*_T_ values based on radiometabolite-corrected plasma activity (*V*_T (AV shunt, Corr)_) to SUV as well as the SUV ratio of tissue uptake to SUV of input function (both IDIF and AV shunt). The use of SUV did not properly relate to the *V*_T (AV shunt, Corr)_ values (*r*^2^ = 0.379, *p* = 0.0011) (Fig. 6a). SUVR values based on SUV of the uncorrected IDIF, SUVR _(IDIF, Uncorr)_, correlated well to *V*_T (AV shunt, Corr)_ (*r*^2^ = 0.907, *p* < 0.0001) (Fig. 6b) while the same approach based on SUVR _(AV shunt, Corr)_ values resulted in a less accurate measurement (*r*^2^ = 0.692, *p* < 0.0001) (Fig. 6c).

## Discussion

The present study described *in vivo* and *in vitro* validation of [^11^C]ITDM specific binding to mGluR1 in the mouse brain, investigated the use of an IDIF for noninvasive kinetic modelling for future longitudinal studies, and characterized pharmacokinetic models for [^11^C]ITDM imaging in mice.

Since, to the best of our knowledge, no *in vivo* validation of [^11^C]ITDM binding to mGluR1 has been reported in the mouse brain, the primary aim of this work was to investigate whether a region devoid of specific [^11^C]ITDM binding existed and thus reference region-based kinetic models could be applied. We demonstrated lack of a suitable reference region in the mouse brain for [^11^C]ITDM. As a first indication, *in vivo* pretreatment with the highly selective mGluR1 antagonist YM-202074 resulted in a significant blockade in the whole brain, primarily in the mGluR1-rich regions (thalamus and cerebellum), but also in low mGluR1 density regions (pons). This evidence was further supported by the *in vivo* displacement study, which clearly showed a manifest decline in [^11^C]ITDM binding following the administration of YM-202074 in the mouse brain, including pons. Based on these findings, we conclude that reference region-based kinetic modelling is not possible for [^11^C]ITDM in mice since no region devoid of specific tracer binding exist [18]. To the best of our knowledge, only one study investigated [^11^C]ITDM binding changes in the mouse brain, quantifying mGluR1 in a model of Huntington’s disease using a reference region-based (pons) kinetic modelling [8]. However, as no *in vivo* validation was performed, the findings based on such analysis should be interpreted with care. Indeed, if neurological disorders, ageing, or their combination have an effect on [^11^C]ITDM specific binding in the pons, this could severely affect reference region-based [^11^C]ITDM quantification and lead to significant misinterpretations of the outcome.

Small animal PET imaging features a relative limited resolution for *in vivo* imaging in relation to the size of the rodent brain; thus, partial volume and spill over effects (PVE) might occur between high contrast regions [19]. Given the anatomical proximity of cerebellum and pons, high and low uptake regions for [^11^C]ITDM respectively, it cannot be excluded that at least a portion of the changes observed in pons might be related to spill in from the cerebellum. In order to investigate this eventuality, we performed *in vitro* blockade of [^3^H]ITDM with different doses of YM-202074, proving blockade of [^3^H]ITDM specific binding occurred in a dose-dependent manner in all regions, pons included. Therefore, since *in vitro* validation supported the *in vivo* findings, we could exclude that the changes observed in the pons were PVE-related.

Noteworthy, when injected in rats, the radiolabelled YM-202074 ([^11^C]YM-202074) *in vivo* metabolism was fairly rapid [20]. Thus, it is conceivable that the extent of *in vivo* blockade measured (79 %) could even be underestimated in relation to the total amount of YM-202074 injected. Nonetheless, as metabolites of YM-202074 do not apply to an *in vitro* setting [20], the agreement observed between the *in vitro* and *in vivo* blockade studies confirmed the lack of suitable reference region. In order to further investigate the extent of *in vivo* specific binding in the pons, we are currently testing an equally potent but more stable compound to block [^11^C]ITDM binding.

The lack of suitable reference region implies the need of measuring an input function in order to perform kinetic modelling of [^11^C]ITDM. However, invasive arterial blood sampling in mice presents several challenges and limitations in the perspective of longitudinal studies due to the small amount of blood collectable and since it is an end of life procedure. Hence, an attractive approach to perform noninvasive quantification is the application of an IDIF [21] to bypass the need for an invasive input function as we previously validated in mice over an atereriovenous shunt for the mGluR5 radiotracer [^11^C]ABP688 [11]. Similar to the findings for [^11^C]ABP688 [11], the IDIF overestimated the tail of the [^11^C]ITDM input function, thus resulted in lower *V*_T_ values. However, *V*_T (IDIF, Uncorr)_ values showed excellent correlation with the *V*_T_ using the metabolite-corrected plasma AV shunt as input function (*V*_T (AV shunt, Corr)_) (*r* = 0.977, *r*^2^ = 0.954, *p* < 0.0001), supporting the applicability of this noninvasive approach for [^11^C]ITDM quantification.

Importantly, mGluRs have been described in the rodent heart [22]. In order to exclude that the higher uptake measured with the IDIF compared to the AV shunt was related to specific binding in myocardiocytes, we compared the IDIF of the same animals during baseline and pretreatment as well as displacement with YM-202074. As for both paradigms no difference in the IDIF was observed (Suppl. Fig. 5, see ESM), the higher values in the IDIF are likely to be due to non-displaceable activity spilling-in from the myocardium, which becomes visible with the decline of blood activity. Indeed, although other organs surround the heart, lungs are characterized by extremely low uptake and liver is not in close proximity to the VOI.

While only intact radiotracer has been described in the rat brain, *in vivo* metabolism of [^11^C]ITDM has been reported in the plasma of rats and non-human primates [7], with nearly 40 % of intact radiotracer 30 min p.i. in parallel with the generation of polar metabolites that do not penetrate the blood-brain barrier. Accordingly, we measured around 31 % of intact radioligand at 30 min p.i. in mice. However, the small blood volume in mice does not allow collection of multiple blood samples for individual metabolite correction with standard techniques. For this reason, we generated a population-based metabolite curve in order to adjust for the peripheral metabolism and to validate the applicability of a noninvasive IDIF over the metabolite-corrected plasma input function. A limitation in the use of a population-based correction is the need of high inter-individual reproducibility; otherwise, under- or overestimations in the quantification may be introduced since the same curve is applied to all the subjects as we recently reported for the radioligand [^11^C]UCB-J [13]. Since correcting the noninvasive IDIF for metabolism did not improve the agreement to *V*_T (AV shunt, Corr)_, we will use the uncorrected IDIF as noninvasive input function. Nonetheless, before studying the radioligand in disease-specific animal models, it is recommended to verify whether animals with different genotype or diseased condition are characterized by altered metabolism.

Kinetic analysis was performed by comparing 1TCM, 2TCM, and Logan plot. According to visual assessment and model selection approaches (AIC and MSC), 1TCM was excluded as it did not fit the data, while both 2TCM and Logan plot proved to be valid alternatives. In addition, time stability of the *V*_T (AV shunt, Corr)_ and *V*_T (IDIF, Uncorr)_ estimations for both 2TCM and Logan plot were investigated. Shortening the scan duration destabilized *V*_T (AV shunt, Corr)_ and *V*_T (IDIF, Uncorr)_ outcomes with both models. This was expected given the fairly gradual brain uptake of [^11^C]ITDM and its slow wash-out profile similar to the findings reported in rats [8].

Finally, we explored whether a different approach simpler than *V*_T (IDIF, Uncorr)_ could be employed to measure [^11^C]ITDM uptake. While SUV was not a reliable measurement, the use of SUVR resulted in more accurate values. Interestingly, SUVR _(IDIF, Uncorr)_ (*r*^2^ = 0.907) performed almost as good as *V*_T (IDIF, Uncorr)_ (*r*^2^ = 0.954) when comparing both measurements to the *V*_T (AV shunt, Corr)_. While this might indicate that a static PET scan 60 to 90 min p.i. could be sufficient, it is important to underline that this approach might still need dynamic PET imaging in order to define the VOI for the IDIF following radioligand injection, unless a different strategy to extract a reliable IDIF is identified. Alternatively, it may be possible that a single blood sample collected during the PET scan could be sufficient to measure blood activity to derive the SUVR _(AV shunt, Corr)_. Yet, such approach appeared less reliable (*r*^2^ = 0.692), invasive, and likely to be sensitive to noise due to the low activity in blood following decay of the radioligand at these later time points p.i.

## Conclusion

We demonstrated that [^11^C]ITDM selectively binds to mGluR1 in mice with no suitable reference region as shown both *in vivo* and *in vitro*. The use of IDIF and a scan duration of 90 min is recommended for accurate noninvasive estimates of [^11^C]ITDM binding. By applying the appropriate kinetic models, [^11^C]ITDM represents a promising tool for studying changes in mGluR1 during comparative studies of ageing and neurological disorders.

## Electronic Supplementary Material

ESM 1 (PDF 1483 kb)
