# Supplementary material for: In vitro and In vivo Assessment of Suitable Reference Region and Kinetic Modelling for the mGluR1 Radioligand [11C]ITDM in Mice
Source: Mol Imaging Biol. 2019 Dec 2;22(4):854–63. doi: 10.1007/s11307-019-01435-1 (PMC7343737; doi:10.1007/s11307-019-01435-1)
Supplement: Supplementary file 1 — (PDF 1483 kb) [file 11307_2019_1435_MOESM1_ESM.pdf]

Electronic Supplementary Material

In vitro and *in vivo* Assessment of Suitable Reference Region and Kinetic Modelling for the mGluR1 Radioligand [^{11}C]ITDM in Mice

Journal: Molecular Imaging and Biology

Daniele Bertoglio[1], Jeroen Verhaeghe[1], Špela Korat[1,2], Alan Miranda[1], Leonie wyffels[1,2], Sigrid Stroobants[1,2], Ladislav Mrzljak[3], Celia Dominguez[3], Longbin Liu[3], Mette Skinbjerg[3], Ignacio Munoz-Sanjuan[3], Steven Staelens[1]

[1]Molecular Imaging Center Antwerp (MICA), University of Antwerp, Wilrijk, Belgium
[2]Department of Nuclear Medicine, Antwerp University Hospital, Edegem, Belgium
[3]CHDI Management/CHDI Foundation, Los Angeles, California, USA

Correspondence to:
Prof. Steven Staelens
Molecular Imaging Center Antwerp (MICA),
Faculty of Medicine and Health Sciences,
University of Antwerp,
Universiteitsplein 1, Wilrijk, Belgium
Tel. +32 03265 2820
Email: steven.staelens@uantwerpen.be

Materials and Methods

Animals

Animals were group-housed in individually ventilated cages under a 12 h light/dark cycle in a temperature- and humidity-controlled environment with food and water *ad libitum*. At least one week of habituation was allowed before the start of the procedures. Six (*n* = 12) and 12 months old (*n* = 18) male C57BL/6J mice from Jackson Laboratories (Bar Harbour, Maine, USA) were used in the study. The 6 monhts old animals were used for the generation of a population-based metabolite curve. Among the 12 months old animals, ten mice were included for baseline PET scans, of which 6 of them were also scanned for either blocking (*n* = 3) or displacement (*n* = 3) studies. The remaining 8 mice were allocated to PET imaging with arteriovenous (AV) shunt.

Radiotracer synthesis

[^{11}C]ITDM synthesis was performed on an automated synthesis module (Carbosynthon I, Comecer, The Netherlands) based on [1]. Briefly, [^{11}C]MeI was added to a precooled (-20°C) reaction vessel containing a solution of stannyl precursor, K_2CO_3, CuCl, $Pd_2(dba)_3$ and $P(o\text{-}tol)_3$ in anhydric DMF and reacted for 5 min at 65°C to synthesize [^{11}C]ITDM, which was subsequently purified with HPLC using an XBridge C18 5µm, 150x10 mm column (Waters XBridge, Belgium) with NaOAC 0.05M pH5.5/EtOH, 50/50 (v/v) as mobile phase. Average radiochemical purity was greater than 99%, and the molar activity was 107.5±28.1 GBq/µmol. [^{3}H]ITDM was synthesized by Pharmaron, UK, with a molar activity of 2.66 GBq/µmol and purity above 99.9%.

PET acquisition

Two Siemens Inveon PET/CT scanners (Siemens Preclinical Solution, Knoxville, USA) were used to acquire the dynamic microPET/Computed tomography (CT) images. Animal preparation was performed as previously described [2-3]. In parallael with the start of the 90 min dynamic PET scan, a bolus of [^{11}C]ITDM was injected using an automated pump (Pump 11 Elite, Harvard Apparatus, USA) over a 12 second interval (1 ml/min). Activity (5.7 ± 2.4 MBq) was injected in a trace dose (0.86±0.33 µg/kg) keeping the cold mass within 2.0 µg/kg as no mass effect could be observed within this limit (Suppl. Fig. 1). PET data were acquired in list-mode format. Following the PET scan, a 10 min 80 kV/500 µA CT scan was performed on the same gantry for attenuation correction and coregistration purposes.

To assess the possibility of a suitable reference region, two treatment studies (blocking and displacement) using the high affinity mGluR1 antagonist YM-202074 (Merck, Germany) were executed. YM-202074 was administered with an i.v. bolus injection of 50 µl with the highest soluble dose (20 mg/kg) in saline. During the first paradigm (blocking study), YM-202074 was injected 2 min before [^{11}C]ITDM injection and the start of the PET scan. In the second paradigm (displacement study), YM-202074 was injected 30 min after [^{11}C]ITDM injection. One animal died during the baseline scan therefore it was excluded from all analysis.

In order to compare the noninvasive IDIF with the gold standard arterial input function, an AV shunt was surgically inserted into the femoral vein and artery prior to the PET scan as we previously described [2]. Once the animal was positioned onto the scanner, the shunt was connected to a peristaltic pump with tubing from the artery led through the Twilite detector [4] and ran in the pump. The tubing coming from the vein was connected on the output line of the pump, together with a second line for tracer

injection. The arteriovenous shunt surgery failed in 2 mice and 1 mouse died during the scan. These animals were omitted from the analysis.

Image reconstruction and processing

Acquired PET data were histogrammed and reconstructed into 39 frames of increasing length (12x10s, 3x20s, 3x30s, 3x60s, 3x150s and 15x300s) using a list-mode iterative reconstruction with proprietary spatially variant resolution modelling in 8 iterations and 16 subsets of the 3D ordered subset expectation maximization (OSEM 3D) algorithm [5]. Normalization, dead time, and CT-based attenuation corrections were applied. PET image frames were reconstructed on a 128x128x159 grid with 0.776x0.776x0.776 mm^3 voxels.

PMOD 3.6 software (Pmod Technologies, Zurich, Switzerland) was used for analysis and processing of the PET data. In order to spatially normalize the PET images, a [^{11}C]ITDM PET template was generated using the 90 min static PET images of the baseline scans, modifying the previously described procedure [3] to [^{11}C]ITDM. Since the blocking and displacement experiments were lacking part of the specific signal, the PET template approach could not be applied. Thus, for these data, individual CT images were spatially normalized to the CT image of the PET template adapting the previously described procedure [3]. All images were visually checked for accuracy following spatial transformation.

During the PET scans with AV shunt, the arterial input function was obtained using a 1 s sampling interval from the whole blood activity derived from the Twilite count detection coupled with the AV shunt. The activity measured with the Twilite was decay and background corrected as well as cross-calibrated with the PET scanner each experimental day. A three-exponential function was fitted to the decaying part of the

input function in order to reduce the noise in the Twilite data. The whole body of the animal was in the microPET scanner's field of view (FOV), hence it was possible to extract the IDIF as a volume-of-interest (VOI) (threshold set to 50% of max) in the lumen of the left ventricle of the heart. Half of the VOI's maximum was found to be the optimal image threshold in order to obtain a stable volume across subjects. The ventricular region was delineated on an early time frame exhibiting maximal activity as previously described [2] and shown in Suppl. Fig. 2.

The [^{11}C]ITDM PET template was generated in the same space of the Waxholm atlas [6], thus VOIs were adapted from this atlas. Regional time-activity curves (TACs) were extracted for striatum, thalamus, hippocampus, cerebellum, and pons.

Metabolite correction

In order to generate a population-based metabolite correction to account for peripheral radiometabolism, we measured parent fractions in a cohort of WT mice (n = 3 per time point) at 5, 15, and 30 min p.i. The procedure was done adapting the previously described methodology [7] to [^{11}C]ITDM. Briefly, mice were injected with the radioligand via the lateral tail vein and blood collected via cardiac puncture at the respective p.i times. Following centrifugation of blood at 2377×rcf for 5 min, both plasma and residual fractions were counted in a gamma counter (Wizard^2, PerkinElmer) in order to determine the plasma-to-whole-blood ratio. Next, equal amounts of ice-cold acetonitrile and 10 µl of cold reference (1 mg/ml) were added to the plasma samples. After another centrifugation at 2377×rcf for 5 min, supernatant was separated from the precipitate and both fractions were counted in the gamma counter to calculate the plasma extraction efficiency (95.8 ± 3.0%). Next, 100 µl of supernatant were loaded onto a pre-conditioned reverse-phase (RP)-HPLC system (Kinetex, 150×4.6mm, 5 µm

HPLC column + Phenomenex security guard pre-column) and eluted with 0.1% TFA in H_20 and acetonitrile (67:33 v/v) buffer at a flow rate of 1.5 ml/min. RP-HPLC fractions were collected at 0.5 min intervals for 8 min and radioactivity was measured in the gamma counter. The radioactivity was expressed as a percentage of the total area of the peaks. Blood spiked *in vitro* with 37 kBq of radiotracer indicated no degradation occurred during the workup (parent = 98.0 ± 0.9%).

In PMOD 3.6 software, average parent fraction values were fitted using a sigmoid curve as this model provided the best fit. This fitted model was then used for population-based metabolite correction and each individual input function (AV shunt or IDIF) was corrected with this parent fraction model as well as for the plasma-to-whole-blood ratio, calculated based on the radiometabolite data. Thus, the metabolite-corrected plasma input function (Corr) was derived and compared to the uncorrected one (Uncorr). The radiometabolite-corrected plasma activity curve derived from the AV shunt measurement was used as gold standard for analysis and assessment of the noninvasive IDIF.

Kinetic modelling

To determine the relative performance of each model to fit the regional PET data using both metabolite-corrected plasma AV shunt as well as IDIF uncorrected as input functions, the goodness-to-fit was calculated using two different approaches: the Akaike Information Criterion (AIC) [8] was applied to 1TCM, 2TCM, and Logan plot, while the Model Selection Criterion (MSC), a modified version of the AIC criterion used in the *Scientist* Software (MicroMath, Saint Louis, Missouri USA), to 1TCM and 2TCM. In addition, values based on 2TCM and Logan obtained with the metabolite-

corrected plasma AV shunt input function ($V_{T\ (AV\ shunt,\ Corr)}$) and noninvasive IDIF ($V_{T\ (IDIF,\ Uncorr)}$) were compared.

Additionally, the time stability of the $V_{T\ (AV\ shunt,\ Corr)}$ and $V_{T\ (IDIF,\ Uncorr)}$ estimations were analysed by repeatidly excluding the last 5 min of PET acquisition from 90 min down to 45 min. Shorter acquisitions were compared to the 90 min PET acquisition, which was considered the reference outcome. Quantification of PET data was considered acceptable when the average percentage difference compared to the 90 min PET acquisition was less than 10% with an inter-individual standard deviation below 5%.

Finally, we explored whether a simplified approach for measumrent of [^{11}C]ITDM could be applied by investigating the standardized uptake values (SUV), and the SUV ratio (SUVR) of target region over the SUV of the input function measured with either the IDIF (SUVR (IDIF, Uncorr)) or plasma metabolite-corrected AV shunt (SUVR (AV shunt, Corr)). All simplified measurements were based on the PET frames from 60 to 90 min.

[^{3}H]ITDM autoradiography

To further evaluate the suitability of a reference region, we investigated the effect of mGluR1 blockade on *in vitro* [^{3}H]ITDM autoradiography. Sections were thawed at room temperature, pre-incubated for 20 min with binding buffer (50 mM Tris-HCl buffer, pH 7.4, containing 2 mM $CaCl_2$ and 1.2mM $MgCl_2$), and dried with a warm airflow. Next, incubation with total binding solution (0.5 nM of [^{3}H]ITDM in binding buffer), non-specific binding solution (0.5 nM of [^{3}H]ITDM + 1 μM of cold ITDM in binding buffer), or blocking solution (0.5 nM of [^{3}H]ITDM + 1 μM or 1 mM of YM-202074 in binding buffer) was performed for 1 hour at room temperature. Sections were washed in 50 mM Tris-HCl buffer on ice, followed by 5 dips into distilled water, and

dried for 2 hours at room temperature. Lastly, slides were exposed on imaging plates (BAS-TR2025, Fujifilm, Japan) for 90 h. A phosphor imager (Fuji FLA-700 image reader) was used to detect radioactivity, which was quantified based on intensity values calculated using tritium standards (American Radiolabeled Chemicals Inc., USA). Regional specific binding was calculated by subtracting nonspecific binding from total binding in triplicate using ImageJ software (National Institute of Health, USA). [^{3}H]ITDM binding was measured in regions of interest, namely striatum, hippocampus, thalamus, pons, and cerebellum, manually drawn on each section.

References

1. Fujinaga M, Yamasaki T, Maeda J, et al. (2012) Development of N-[4-[6-(isopropylamino)pyrimidin-4-yl]-1,3-thiazol-2-yl]-N-methyl-4-[11C]methylbenzamide for positron emission tomography imaging of metabotropic glutamate 1 receptor in monkey brain. J Med Chem 55:11042-11051.
2. Verhaeghe J, Bertoglio D, Kosten L, et al. (2018) Noninvasive Relative Quantification of [(11)C]ABP688 PET Imaging in Mice Versus an Input Function Measured Over an Arteriovenous Shunt. Front Neurol 9:516.
3. Bertoglio D, Verhaeghe J, Kosten L, et al. (2018) MR-based spatial normalization improves [18F]MNI-659 PET regional quantification and detectability of disease effect in the Q175 mouse model of Huntington's disease. PLoS One 13:e0206613.
4. Alf MF, Wyss MT, Buck A, Weber B, Schibli R, Kramer SD (2013) Quantification of brain glucose metabolism by 18F-FDG PET with real-time arterial and image-derived input function in mice. J Nucl Med 54:132-138.
5. Miranda A, Glorie D, Bertoglio D, et al. (2019) Awake (18)F-FDG PET Imaging of Memantine-Induced Brain Activation and Test-Retest in Freely Running Mice. J Nucl Med 60:844-850.
6. Johnson GA, Badea A, Brandenburg J, et al. (2010) Waxholm space: an image-based reference for coordinating mouse brain research. Neuroimage 53:365-372.
7. Bertoglio D, Verhaeghe J, Miranda A, et al. (2019) Validation and noninvasive kinetic modeling of [(11)C]UCB-J PET imaging in mice. J Cereb Blood Flow Metab:271678X19864081.
8. Akaike H (1974) A new look at the statistical model identification. IEEE Transactions on Automatic Control 19:716-723.

Supplementary figures

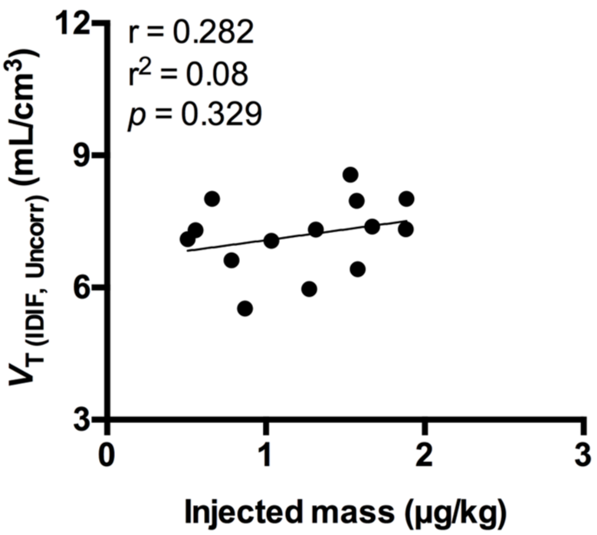

Supplementary Fig. 1. Relationship between injected mass and cerebellar $V_{T\ (IDIF,\ Uncorr)}$ values. No injected mass effect (r = 0.282, *p* = 0.329) was present at the mass doses used. *n* = 14.

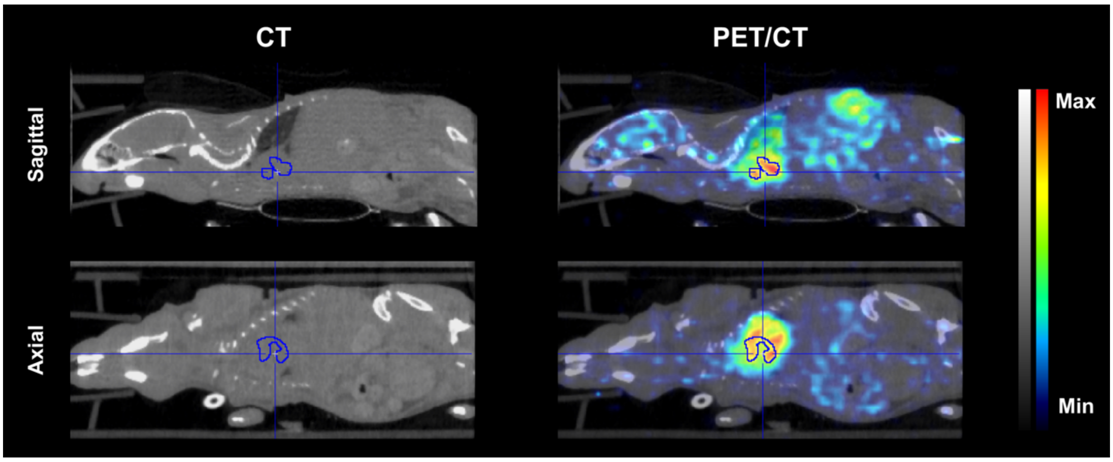

Supplementary Fig. 2. Representative cardiac volume-of-interest (VOI) for the measurement of the image-derived input function (IDIF). VOI for IDIF extraction was obtained by generating a sphere (3.5 mm in diameter) in the heart on the CT image. Next, the early 10s PET frame exhibiting maximal activity in the heart was selected and the VOI was generated by setting a threshold to 50% of max activity in the VOI. The IDIF VOI was obtained as shown in the figure and the IDIF was extracted from the dynamic PET acquisition.

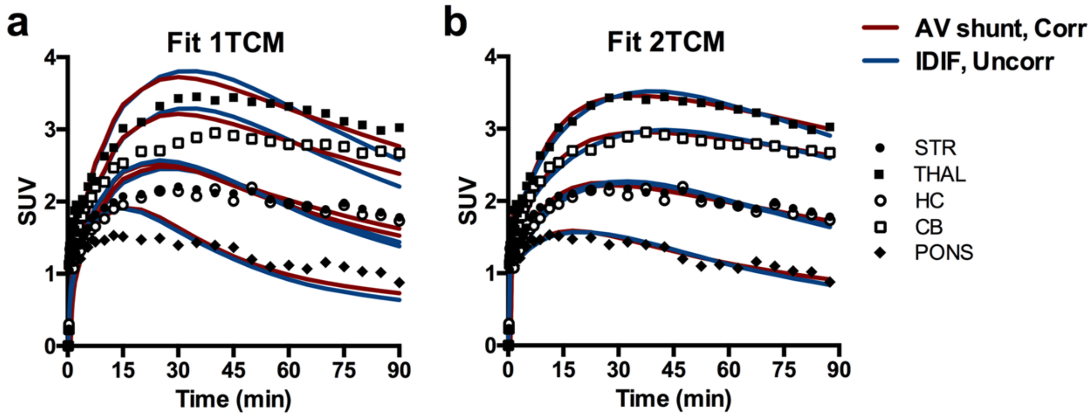

Supplementary Fig. 3. Representative fitting of kinetic models. (a) One-tissue comparmetal model (1TCM) and **(b)** two-tissue comparmetal model (2TCM) ~~of~~ were fitted to the time-activity curves ~~during baseline scan~~ based on either the image-derived input fuciton (IDIF, Uncorr) or metabolite-corrected plasma AV shunt (AV shunt, Corr) as input function. STR = striatum, THAL = thalamus, HC = hippocampus, CB = cerebellum.

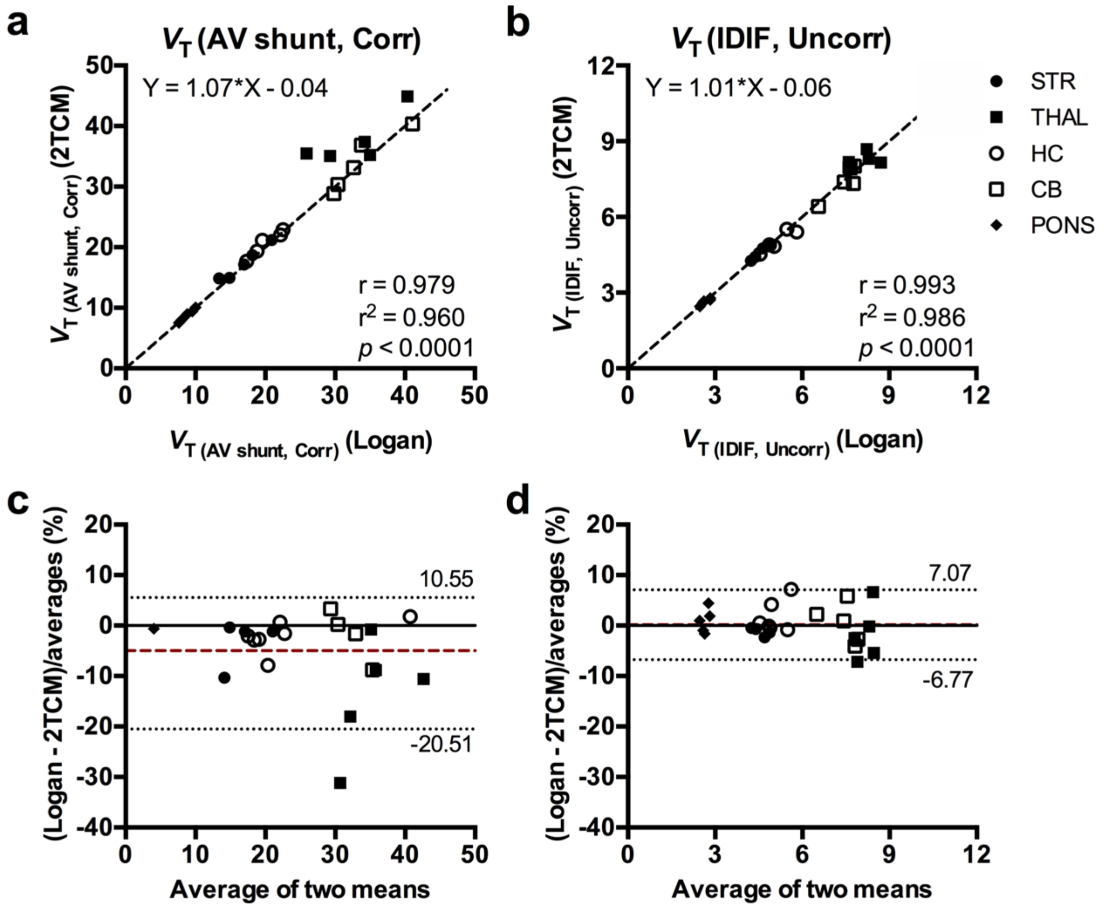

Supplementary Fig. 4. Comparison of V_T values obtained using 2TCM versus Logan graphical analysis. Kinetic modelling based on 2TCM and Logan plot based on 90 min acquisition showed excellent agreement for both the $V_{T\ (AV\ shunt,\ Corr)}$ **(a)** and $V_{T\ (IDIF,\ Uncorr)}$ **(b)** values. Dashed line depicts the identity line. Bland-Altman plots were applied to compare 2TCM and Logan estimations based on the $V_{T\ (AV\ shunt,\ Corr)}$ **(c)** and $V_{T\ (IDIF,\ Uncorr)}$ **(d)** values. Red dashed line represents the bias between the 2 approaches, while the dotted lines denotes the 95% limits of agreement. *n* = 5. STR = striatum, THAL = thalamus, HC = hippocampus, CB = cerebellum.

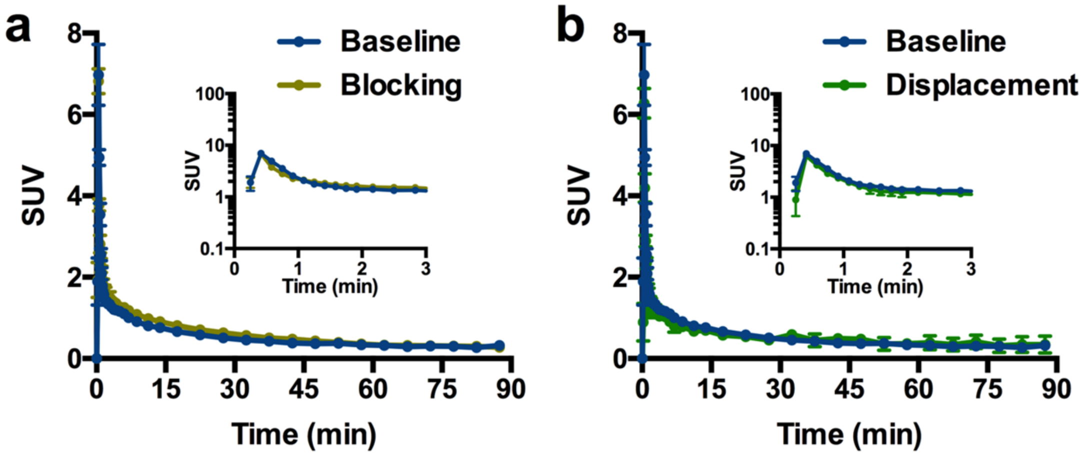

Supplementary Fig. 5. Comparison of [^{11}C]ITDM image-derived input function (IDIF) during baseline, blocking, and displacement studies. No difference in the IDIF was visible during blocking **(a)** and displacement **(b)** compared to the baseline scan. *n* = 3 per group. Data are mean ± standard error mean.

Supplementary Tables

Supplementary Table 1. Akaike information criterion (AIC) values for model selection in each investigated brain region determined using IDIF ($V_{T\ (IDIF,\ Uncorr)}$) and metabolite-corrected plasma AV shunt ($V_{T\ (AV\ shunt,\ Corr)}$) input functions based on PET acquisition of 90 min. The preferred model is the one with the lowest AIC value. n = 5 ~~9~~.

Region	Akaike information criterion (AIC)					
	$V_{T\ (IDIF,\ Uncorr)}$			$V_{T\ (AV\ shunt,\ Corr)}$		
	1TCM	2TCM	Logan plot	1TCM	2TCM	Logan plot
	Mean (%COV)	Mean (%COV)	Mean (%COV)	Mean (%COV)	Mean (%COV)	Mean (%COV)
**Striatum**	108.3 (7.2)	41.2 (11.7)	125.2 (12.7)	95.1 (27.1)	63.4 (72.9)	146.7 (10.2)
**Thalamus**	99.4 (6.6)	41.4 (49.2)	115.5 (27.3)	80.2 (30.5)	59.2 (57.1)	160.8 (10.3)
**Hippocampus**	111.9 (10.0)	37.5 (21.7)	119.7 (4.3)	96.5 (31.2)	64.6 (78.9)	150.6 (5.0)
**Cerebellum**	103.9 (5.4)	31.5 (33.2)	118.6 (8.7)	80.1 (31.8)	40.3 (128.0)	148.4 (8.35)
**Pons**	132.7 (10.4)	68.9 (20.6)	132.5 (11.5)	134.4 (17.0)	97.2 (41.4)	154.1 (9.9)

1TCM = 1-tissue compartmental model, 2TCM = 2-tissue compartmental model, COV = coefficient of variation.

Supplementary Table 2. Model Selection Criterion (MSC) values for model selection in each investigated brain region determined using both IDIF ($V_{T\ (IDIF,\ Uncorr)}$) and metabolite-corrected plasma AV shunt ($V_{T\ (AV\ shunt,\ Corr)}$) input functions based on PET acquisition of 90 min. The preferred model is the one with the highest MSC value. *n* = 5 ~~9~~.

Region	Model Selection Criterion (MSC)			
	$V_{T\ (IDIF,\ uncorr)}$		$V_{T\ (AV\ shunt,\ Corr)}$	
	1TCM	2TCM	1TCM	2TCM
	Mean (%COV)	Mean (%COV)	Mean (%COV)	Mean (%COV)
**Striatum**	0.85 (33.1)	2.65 (13.3)	1.20 (54.3)	2.06 (56.8)
**Thalamus**	1.48 (12.2)	3.05 (11.7)	2.00 (26.1)	2.57 (26.8)
**Hippocampus**	0.81 (26.3)	2.80 (6.3)	1.22 (60.9)	2.08 (62.9)
**Cerebellum**	1.23 (15.2)	3.17 (8.1)	1.87 (31.5)	2.94 (43.1)
**Pons**	0.04 (455.2)	1.76 (19.4)	0.01 (50.2)	1.01 (93.5)

1TCM = 1-tissue compartmental model, 2TCM = 2-tissue compartmental model, COV = coefficient of variation.
